# Drilling Predation on Serpulid Polychaetes (*Ditrupa arietina*) from the Pliocene of the Cope Basin, Murcia Region, Southeastern Spain

**DOI:** 10.1371/journal.pone.0034576

**Published:** 2012-04-05

**Authors:** Jordi Martinell, Michał Kowalewski, Rosa Domènech

**Affiliations:** 1 Institut de Recerca de la Biodiversitat and Facultat de Geologia, Universitat de Barcelona, Barcelona, Spain; 2 Department of Geosciences, Virginia Tech, Blacksburg, Virginia, United States of America; Institut de Biologia Evolutiva - Universitat Pompeu Fabra, Spain

## Abstract

We report quantitative analyses of drilling predation on the free-living, tube-dwelling serpulid polychaete *Ditrupa arietina* from the Cope Cabo marine succession (Pliocene, Spain). Tubes of *D. arietina* are abundant in the sampled units: 9 bulk samples from 5 horizons yielded ∼5925 specimens of *D. arietina*. Except for fragmentation, tubes were well preserved. Complete specimens ranged from 3.1 to 13.4 mm in length and displayed allometric growth patterns, with larger specimens being relatively slimmer. Drilled *Ditrupa* tubes were observed in all samples. Drillholes, identified as *Oichnus paraboloides*, were characterized by circular to elliptical outline (drillhole eccentricity increased with its diameter), parabolic vertical profile, outer diameter larger than inner diameter, penetration of one tube wall only, narrow range of drill-hole sizes, and non-random (anterior) distribution of drillholes. A total of 233 drilled specimens were identified, with drilling frequencies varying across horizons from 2.7% to 21% (3.9% for pooled data). Many tube fragments were broken across a drillhole suggesting that the reported frequencies are conservative and that biologically-facilitated (drill-hole induced) fragmentation hampers fossil preservation of complete serpulid tubes. No failed or repaired holes were observed. Multiple complete drillholes were present (3.9%). Drilled specimens were significantly smaller than undrilled specimens and tube length and drill-hole diameter were weakly correlated. The results suggest that drillholes were produced by a size-selective, site-stereotypic predatory organism of unknown affinity. The qualitative and quantitative patterns reported here are mostly consistent with previous reports on recent and fossil *Ditrupa* and reveal parallels with drilling patterns documented for scaphopod mollusks, a group that is ecologically and morphologically similar to *Ditrupa*. Consistent with previous studies, the results suggest that free-dwelling serpulid polychaetes are preyed upon by drilling predators and may provide a viable source of data on biotic interactions in the fossil record.

## Introduction

This study documents drilling predation patterns on the free-living, tube-dwelling serpulid polychaete *Ditrupa arietina* from the Pliocene of southeastern Spain. Free-living serpulids occur in many marine benthic ecosystems and are also known from the fossil record. Thus, although currently underutilized, the shell-bearing serpulids are potentially an important source of quantifiable data on biotic interactions between polycheates and drilling predators, past and present.

Drilling predators are phylogenetically diverse, geographically widespread, and often abundant in marine ecosystems [1], [2], [3]. Also, drillers have produced a rich fossil record of ecological interactions, with quantifiable data spanning from the Ediacaran [4], [5] to the Holocene [6], [7], [8]. This rich fossil record can be used to study many paleobiological questions, including behavior of predators [9], [10], [11], [12], [13], biotic interactions in individual fossil assemblages [14], [15], or evolutionary ecological trends across assemblages over multiple spatial and temporal scales [1], [3], [16], [17], [18], [19], [20], [21].

In recent years, the concerted efforts of paleontologists and ecologists have advanced considerably our knowledge of drilling predation/parasitism and demonstrated that drilling is employed by many groups of predators/parasites and that many shell-bearing groups of prey/host are drilled (see compilations [1], [2], [22], [23] and references therein). However, whereas several major groups have been explored extensively – including, in particular, mollusks [1], [23], [24], brachiopods [21], [25], [26], [27], [28], [29], and echinoderms [30], [31], [32], [33], [34], [35] – many viable prey (or host) groups have remained understudied despite their potential as a source of quantitative ecological data. Shell-bearing serpulid polychaetes offer a good case example of this problem.

The free-living, shell-bearing polycheates should be an attractive target for paleoecological studies on drilling predation given that (1) they are frequent and locally abundant in the present-day ecosystems [36]; (2) they can occur in great abundance in fossil assemblages [37], [38], [39], [40], [41]; and (3) drilling in modern populations has been reported previously [42], [43]. Yet, we know of only a handful of studies that document drillholes in fossil *Ditrupa* in some detail. These include reports of the Eocene *Ditrupa* from the Paris Basin [44] and the Pliocene *D. arietina* and *D. brevis* from southern Italy [45]. Neither represents an in-depth quantitative study of drilling predation. A recent study of Klompmaker [46] on drillholes in the Pliocene *D.* cf. *arietina* is the first detailed paleontological report known to us. In addition, drillholes in serpulid polychaetes have been reported from the Paleocene of Belgium and Netherlands [47]. A similar dearth of information exists in the ecological literature dedicated to present-day marine benthic ecosystems. Despite the fact that *D. arietina* (Müller, 1776) [48] is abundant in many subtidal soft-bottom communities of the Mediterranean Sea and the northeastern Atlantic Ocean, a detailed study of drillholes in *Ditrupa* has been published only recently [43], [49]. Morton and Harper [43] provide diverse qualitative and quantitative data that represent a valuable baseline for paleontological studies because *D. arietina* (and morphologically similar species) are known from multiple localities in the Cenozoic fossil record [41], [44], [45].

**Figure 1:**
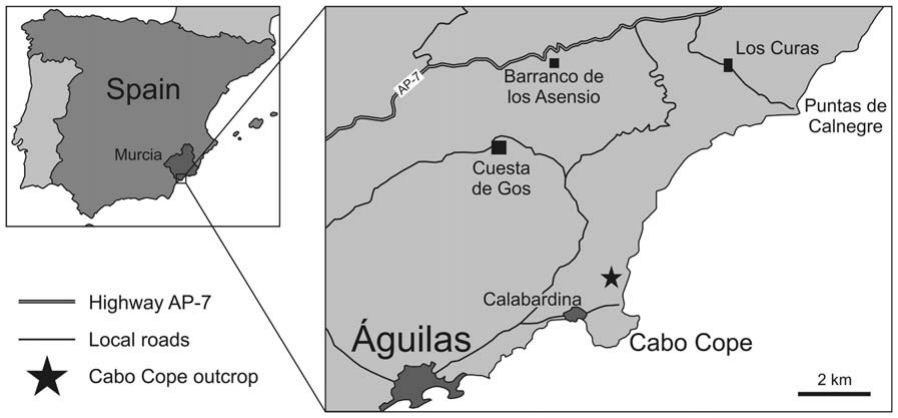
Geographic location of the Cabo Cope outcrop (Murcia Region, SE Spain).

This study documents drilling predation on *D. arietina* from the Pliocene of the southeastern Spain (Fig. 1). Specifically, we present qualitative and quantitative analyses of a series of bulk samples collected vertically along the Cope Cabo outcrop (Murcia Region, SE Spain), which represents a well-developed succession of Plio-Pleistocene marine and continental sediments.

## Materials and Methods

### Study Area and Geological Setting

All samples were collected from the Cabo Cope outcrop, located ∼2.5 km south of Calabardina, a suburban part of the town of Águilas, Murcia Region, southeastern Spain (Fig. 1). All necessary permits were obtained for the described field studies. These include: (1) Permission for paleontological and sedimentological prospecting granted by the Historical Heritage Service of the Murcia Region Government, Spain (ref. CCJD/DGBABC/SPH n° 676/2008) and issued to J. Martinell and C. Dabrio; and (2) Consent from the “Owners Association of the Marina de Cope Action of Regional Interest”, Marina de Cope, Murcia Region, Spain.

The sampled outcrop is located in the Neogene Cope Basin, a small area (<40 km^2^) situated east of the inner part of the Bethic Cordillera. The elevated parts of the basin topography are primarily formed by Jurassic limestone and dolomite of the Malaguide Complex of the Bethic Cordillera.

Viseras et al. [50] divided Neogene sedimentary rocks of the eastern Bethic Cordillera into 6 units. The two uppermost of those units (Units 5 and 6), exposed in the Cope Basin area, represent one of the most complete successions of the Pliocene-Pleistocene marine and continental sediments in the southwestern Mediterranean [50]. The basal part of the sequence – known only from subsurface, as a thin package of Pliocene continental deposits – is overlain by blue clay. The clay, assigned to *Globorotalia puncticulata-margaritae* biozone [51], likely represents a deep water deposit [51]. It gradually transitions upward into a sandy calcarenitic facies, suggestive of a shallowing-upward, regressive trend [51]. Following Bardají [51], the calcarentic facies have been assigned to the middle-late Pliocene, although palaeomagnetic samples collected from the nearby Cabo Cope outcrop suggest that the uppermost strata may be of the early Pleistocene age [52].

**Figure 2:**
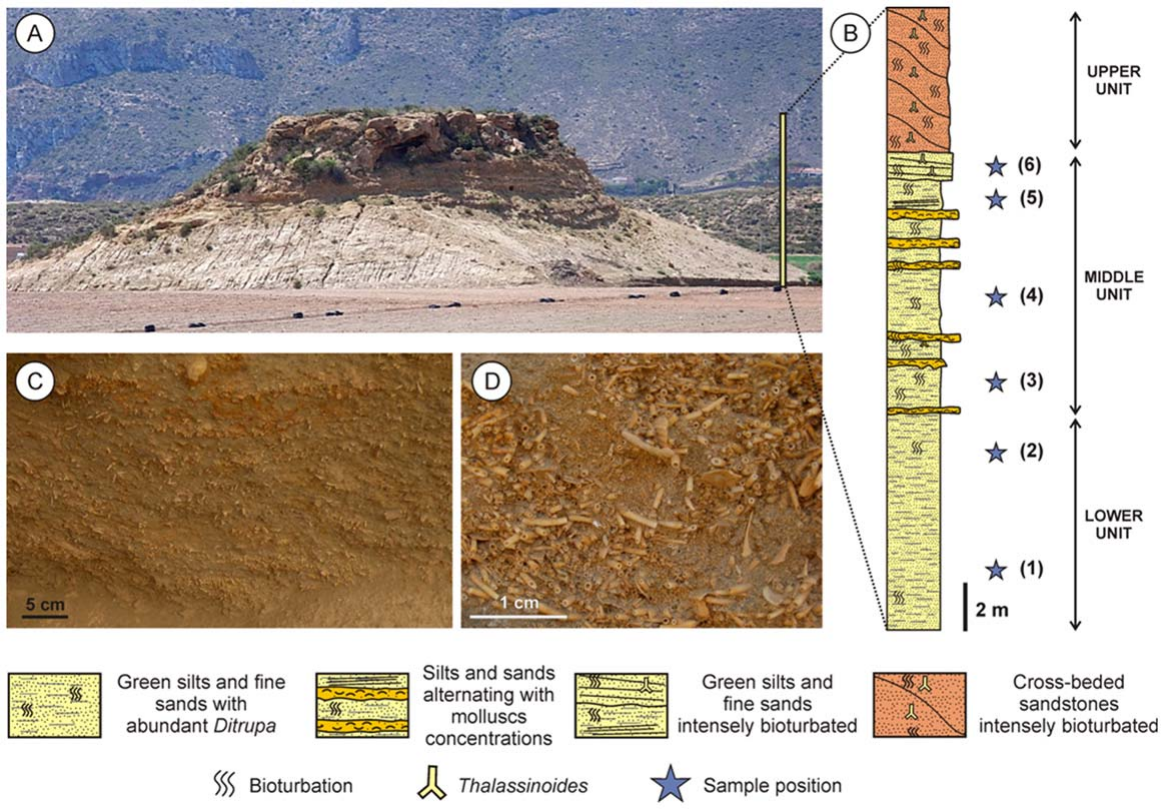
Stratigraphy and biostratinomy of the Cabo Cope outcrop. (a) A panoramic view of the northwestern wall of the outcrop; (b) A schematic stratigraphic column of the three informal units distinguishable in the outcrop (stratigraphic position of samples indicated with stars); (c) A close-up of matrix-supported skeletal accumulations dominated by abundant tubes of *Ditrupa arietina* (Lower Unit); (d) A close-up of a monospecific concentration of *D. arietina* (Middle Unit).

Cabo Cope is a small oval-shaped hill (∼70 m long, ∼50 m wide, and ∼30 m high relative to the surrounding basin floor) located in the middle of an agricultural plain (Fig. 2a). The hill represents an erosional remnant of a lutitic platform that developed in the Cope Basin area in the Pliocene. The surrounding areas are covered by continental Quaternary deposits. The basal part of the hill is dominated by fine sandy clay, which transitions upward into unlithified to poorly lithified sand that is increasingly lithified toward the top of the outcrop. The stratigraphic succession can be informally divided into three units, referred to here as the “lower”, “middle”, and “upper”, respectively (Fig. 2b). The lower unit (0–10.5 m) consists of poorly sorted green mud and sand, with relatively abundant macrofauna (primarily annelids, brachiopods, and pectinids). The unit is bioturbated (*Thalassinoides*) and most of the original sedimentary structures appear to have been obliterated, except for some thin streaks of fine bioskeletal material still discernible in the outcrop. The middle unit (10.5–20 m) is composed of greenish silt and fine sand, punctuated by heavily bioturbated horizons (*Ophiomorpha*, *Thalassinoides*). The macrofauna consists of annelids, barnacles, gastropods, oysters, pectinids, venerid bivalves, brachiopods, bryozoans, and echinoids. Up to seven fossiliferous levels with distinct fossil associations can be discerned vertically within the middle unit. The upper unit (20–30 m) includes two distinct subunits. The lower subunit consists of coarse sandstone with intense bioturbation (*Thalassinoides*) and large-scale low-angle cross-stratification. The upper subunit consists of coarse and very coarse yellow-to-light-brown sandstone. The upper subunit is intensely bioturbated (*Thalassinoides* and, possibly, some *Ophiomorpha*) and contains dispersed valves and valve fragments of pectinids. The bedding plane forming the top surface of the Cabo Cope hill is heavily bioeroded, including *Circolites*, *Gastrochaenolites*, and some sinuous traces of unknown origin. This extensive bioerosion suggests that the surface represented a rocky coast at some point during the Quaternary.

All samples collected in this study came from the Cabo Cope outcrop (Fig. 2b) and represent the unlithified sediments of the lower and middle units. These units represent the middle-to-late Pliocene deposits of the sandy calcarenitic facies. Table 1 summarizes a list of macrofossil taxa collected in previous surveys of the Cabo Cope conducted by our research group.

**Table 1:** A presence-absence list of macrofossil taxa documented from the three informal stratigraphic units of the Pliocene succession of the Cabo Cope outcrop.

Taxa	Informal stratigraphic unit		
	Lower	Middle	Upper
Coelenterata			
Unidentified scleractinians		•	
Annelida (Polychaeta)			
*Ditrupa arietina* (O.F. Müller, 1776)	•	•	
Arthropoda (Cirripedia)			
*Balanus concavus* Bronn, 1831		•	
*Balanus stellaris* (Brocchi, 1789)		•	
Mollusca (Bivalvia)			
*Chlamys multistriata* (Poli, 1795)		•	
*Aequipecten opercularis* (Linnaeus, 1758)	•	•	
*Aequipecten scabrella* (Lamarck, 1819)	•	•	
*Pecten jacobaeus* (LInnaeus, 1758)		•	•
*Macrochlamys latissima* Brocchi, 1814		•	
*Amussium cristatum* (Bronn, 1827)		•	
*Flabellipecten flabelliformis* (Brocchi, 1814)		•	
*Ostrea lamellosa* Brocchi, 1814		•	•
*Ostrea virleti* Deshayes, 1832		•	
*Anomia ephippium* Linnaeus, 1758	•	•	
*Spondylus crassicosta* (Lamarck, 1819)		•	
*Laevicardium* sp.		•	
Unidentified venerids	•	•	
Mollusca (Gastropoda)			
*Amalthea* aff. *acuta* (Quoy & Gaimard, 1824)		•	
(?) *Antisabia* sp.		•	
*Epitonium algerianum* (Weinkauff, 1866)		•	
*Epitonium clathratulum* (Kanmacher, 1798)		•	
*Epitonium proximus* (De Boury, 1890)		•	
*Cirsotrema lamellosum* (Brocchi, 1814)		•	
*Cirsotrema pumiceum* (Brocchi, 1814)		•	
Brachiopoda			
*Ancistocrania abnormis* (Defrance in Hoeninghaus, 1828)		•	
*Aphelesia* cf. *margineplicata* (Philippi, 1844)		•	
*Maltaia pajaudi* García Ramos, 2006	•	•	•
*Megathiris detruncata* (Gmelin, 1791)		•	
*Megerlia truncata* (Linnaeus, 1767)		•	
Bryozoa			
*Manzonella exilis* (Manzoni, 1869)		•	
Echinodermata (Echinodiea)			
*Schizaster* sp.		•	
*Echinolampas* aff. *hoffmani*		•	
*Spatangus* sp.		•	
*Arbacina romana* (Merian in Desor, 1858)		•	

### Taxonomic Identity and Morphology of Sampled Serpulid Polychaetes

Numerous annelid tubes collected from various stratigraphic levels of the Cabo Cope succession were examined under microscope. All analyzed serpulid specimens were identified as *Ditrupa arietina* (Müller, 1776) [48], an extant infaunal, free-living suspension-feeding serpulid polychaete that is widespread and abundant today in the Mediterranean Sea [36], [53], [54], [55], [56] and along the coasts of the eastern Atlantic, from Iceland to Senegal [49], [57]. This species is considered an indicator of unstable sea floors [39], [58], tolerant of high quantities of suspended inorganic matter, and capable of flourishing under turbid conditions [37].

The genus *Ditrupa* is characterized by a distinct calcitic exoskeleton in the form of a tusk-shaped tube. This distinct morphology often causes *Ditrupa* to be misidentified as a scaphopod mollusk [45], [59]. In *D. arietina*, the tube is slim, thin-walled, slightly-to-moderately arcuate, circular in cross-section, and opened on both ends. The tube diameter increases anteriorly and a slight globular widening is typically present around their anterior end. The external tube surface tends to be smooth, although variably pronounced concentric growth lines are often discernible. In present-day specimens, the inner shell surface tends to be lighter than the outer one. Tubes secreted by *D. arietina* are relatively small. In the western Mediterranean specimens can reach over 35 mm in tube length, although variation in size structure is high among populations and through seasons [36], [60]. In the eastern Atlantic, the maximum tube length of 23 mm has been reported from the Azorean platform [43]. All specimens examined in this study share the above morphological characteristics, including relatively small body size (<25 mm in length).

### Stratigraphic Distribution and Taphonomy of *Ditrupa arietina*

Invertebrate fossils are abundant throughout the Cabo Cope stratigraphic succession and occur primarily as localized shell concentrations. In terms of taxonomic composition two main types can be distinguished: (1) annelid concentration dominated by *D. arietina* (Fig. 2d) and (2) diverse pectinid concentrations (7 species from 6 different genera; see Table 1). In terms of biostratinomy, four distinct types of shell concentrations can be distinguished: (A) Irregular small lenses, with a maximum dimension between 7 and 25 cm; (B) Infills of vertical burrows (*Ophiomorpha*); (C) Small residual lags lining basal parts of depressions that may record either biological or physical erosion; and (D) Thin, only a few millimeter thick, but laterally widespread shelly horizons. Tubes and tube fragments of *D. arietina* appear chaotically oriented in the type A and B concentrations and directionally oriented (parallel to bedding) in the type C and D concentrations. The four types of concentrations are observed in the lower and middle units, although irregular lenses and burrow infills appear to be more common in the lower unit. In all annelid-dominated shell concentrations, regardless of their type, specimens of *D. arietina* are extremely abundant, although most of the fossils are tube fragments, and not complete specimens.

In addition, to those four-type of annelid concentrations, tubes of *D. arietina* also occur dispersed in sediments throughout both the lower and the middle unit (Fig. 2c). Even in these less fossiliferous sediments, the annelid tubes are still very abundant. However, comparing with shell concentrations, they are less likely to have been altered and sorted by biostratinomic processes. Thus, they are more suitable for quantitative sampling and more likely to yield complete specimens. Consequently, all but one sample used in this study (see below for details) were obtained from sediments with dispersed fossils and shell concentrations were avoided during sampling.

### Sampling and Sample Processing

A total of 11 bulk samples were collected from 6 horizons (stratigraphic levels) of fine-grained sands exposed in the lower and middle parts of the Cabo Cope section (Fig. 2b). Nine out of 11 samples (representing 5 out of the 6 sampled horizons) yielded notable numbers of *D. arietina* specimens (n>50). The remaining two samples are not included in this study. Of the 9 *Ditrupa*-rich samples, four were obtained from a single horizon to assess within-horizon spatial variations in paleoecological and taphonomic patterns. These four samples (1-0, 1-1, 1-2, and 1-3) came from the lowermost sampling level (referred here to as ‘Horizon 1’). Two samples (2-0, 2-1) were taken from the second lowermost horizon (Horizon 2), whereas the successive Horizons 3, 4, and 5 each is represented by a single sample, samples 3-0, 4-0, and 5-0, respectively (see Fig. 2b for the exact stratigraphic position of the horizons).

Each sample was collected using the same protocol. A one bag of sediment was acquired from a single site evaluated in the field as sedimentologically and paleontologically representative of a given Horizon. The only exception is sample 2-1, which was taken from a small, sharply defined lens of bioclastic material representing an exceedingly fossiliferous part of the horizon that may have been biologically concentrated by a large bioturbating predator (e.g., a feeding ray). While the samples were comparable in size, their weights varied slightly. To standardize samples volumetrically, all samples were reduced in volume, by successive removal of a small amount of the sediment, until their weights matched the weight of the smallest of the samples (207 g). Because sample weight exceeded 207 g by small amounts only for all samples and because the removal had been done before sediment was sieved, this protocol did not induce substantial loss of material and is unlikely to have introduced any substantial bias. Thanks to this minor mass adjustment, all samples were standardized to represent the same amount of sampled sediment prior to sieving and specimen sorting. Subsequently, all samples were sieved using two mesh sizes (0.5 and 1 mm). The resulting material was then sorted to separate all specimens of *Ditrupa arietina*. The total weight of all specimens was recorded separately for the 0.5–1 mm and >1 mm size fractions.

### Specimen Analysis

Because all specimens were identified as *D. arietina* (see above), quantitative analyses are simplified as data need not to be subdivided into multiple taxa.

Specimens were examined under binocular microscope and morphometric measurements were obtained using the Leica LAS Software version 3.5 (2009) used to process specimen images captured with the Leica DFC 426 digital camera attached to the LEC Leica MZ6 microscope. The measurement precision was +/− 0.01 mm. Because the processed samples yielded many thousands of specimens (mostly fragments), it was not viable to obtain measurements exhaustively for all individuals. Consequently, the following protocol was employed. First, all drilled specimens (i.e., fragments or complete tubes that included at least one unquestionable drillhole) were separated and measured (Table S1). In addition, a random set of 30 undrilled specimens was separated for each sample (a total of 270 specimens) and measured (Table S1). The specimens were selected from each fraction separately proportionally to percent weight of the fraction (for example, if >1 mm fraction represented 96% of all material, 29 out of 30 specimens were selected from >1 mm fraction and 1 specimen was selected from 0.5–1 mm fraction.

**Figure 3:**
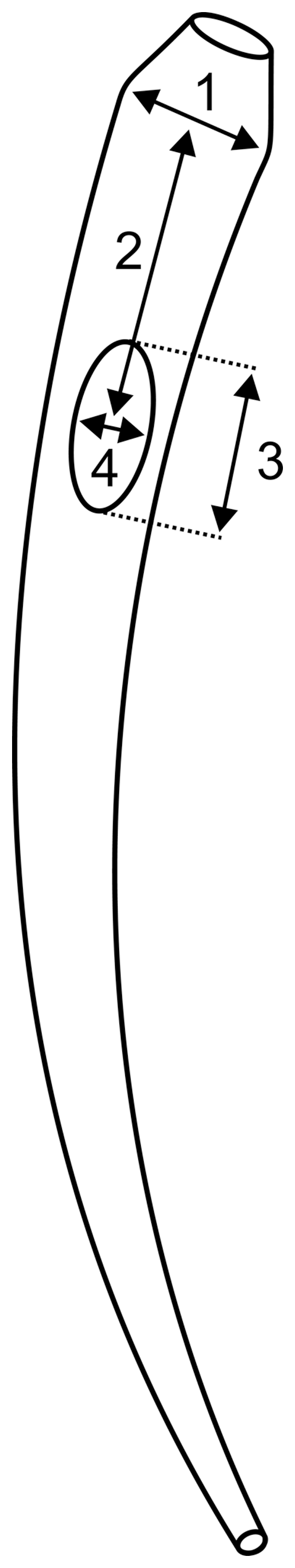
A schematic summary of biometric measurements used in this study for *Ditrupa* tubes and tube fragments. (1) Maximum tube diameter; (2) Relative drillhole location; (3) Maximum drillhole diameter; (4) Minimum drillhole diameter.

For the 270 undrilled specimens, the maximum width of the tube was measured (note that this measurement estimates the actual maximum width of the specimens only in the case of complete specimens or fragments that preserve the anterior endpoint of the tube) (Fig. 3). In addition, the additional 28 specimens, identified as complete (i.e., preserving both the posterior and anterior endpoints of the tube), were separated from the remaining material and measured in terms of both maximum tube width and total tube length (Table S2).

For each drilled specimen, the maximum width of the tube, the maximum drillhole diameter, and minimum drillhole diameter were recorded. For specimens with multiple drillholes, all drillholes were measured. In addition, distance from the anterior end to the center of the drillhole was measured for drilled specimens with the terminal part of the tube preserved (i.e., those for which the true total specimen length could be meaningfully estimated) (Fig. 3).

Because specimens are overwhelmingly dominated by tube fragments, it is difficult to provide realistic estimates of the total number of specimens. Consequently, we employed an indirect approach, in which the total number of specimens in a sample was estimated from the total weight of all tubes and tube fragments in that sample divided by the estimated weight of an average complete specimen. Note here that an alternative method, designed for sediment-filled and/or encrusted tubes and tube fragments (a problem which does not affect the specimens studied here), was proposed recently by Klompmaker [46]). To estimate average specimen weight, the individual weights were measured for 30 complete specimens, which were split into two groups (15 larger and 15 smaller specimens, respectively) and weighed separately. The larger specimens weighed 0.17 g (mean specimen weight of 0.011 g). The set of smaller specimens weighed 0.02 g (mean specimen weight 0.001 g). An average of the two estimates (the mean estimated specimen weight = 0.006 g), which is a mathematical equivalent of the mean computed for all 30 specimens analyzed together, is used in this paper to estimate total numbers of complete specimens represented in the samples. Because fragments do not allow for estimating the actual length of specimens from which they were derived (and thus, we do not know the shape of the underlying size frequency distributions of the sampled population), it is difficult to evaluate if the mean estimated specimen weight reported above is an accurate proxy for the true average specimen weight. Therefore, to bracket our estimates, we also report the maximum frequency estimate derived by using the mean weight value for larger specimens, an approach which is almost certain to grossly underestimate the number of specimens, and thus, grossly overestimate drilling frequency. Conversely, we use the minimum frequency estimate derived by using the mean weight value for smaller specimens, an approach which is almost certain to grossly overestimate the number of specimens, and thus, grossly underestimate drilling frequency. The actual drilling frequency is expected to fall within that bracket, with mean specimen weight being the best available, even if imperfect, estimate of the actual drilling frequency. All weight measurements were acquired using the COB05 C-200-SX balance. The analytical precision was tested directly by reweighting 10 times a set of 10 specimens (standard deviation = 0.0052 g) and also reweighting 10 times a single compete specimen (standard deviation = 0.0042 g). Thus, those two tests consistently indicated the analytical precision of ∼0.005 g.

Quantitative data have been analyzed using both parametric and non-parametric statistical methods. The *a priori* assumed significance level of α = 0.05 is used in all statistical decisions below. For multiple tests, a Bonferroni correction has been applied to correct α (α_B_ = 0.05 divided by the number of simultaneous tests). Because in most cases tests are partly dependent, the correction is conservative. Statistical analyses have been performed using SAS/STAT procedures, custom-written codes in SAS/IML, and PAST.

Specimen-level measurements used in this study are provided as supplementary online materials (Tables S1, S2).

## Results

A total of 9 samples from 5 horizons are included in this analysis. They represent ∼5925 complete specimens, as estimated using the fragmentation-corrected approach discussed above using the mean estimated specimen weight (Table 2). When the mean weight of small specimens is used, the maximum number of sampled specimens is estimated at ∼33098. Conversely, when the mean weight of large specimens is used, the minimum number of sampled specimens is estimated at ∼3310 specimens. Multivariate numerical measurements (Table S1) were collected for 503 specimens (mostly fragments), including all drilled specimens (found by exhaustive screening of samples; *n* = 233) and additional 270 randomly selected undrilled specimens (30 specimens per sample).

**Table 2:** Summary of sampling information and resulting data for the 9 samples used in this study.

Hor-izon	Sam-ple	Total weight of *Ditrupa* specimens [g]	Weight of >1 mm *Ditrupa* fraction [g]	Weight of 0.5–1 mm *Ditrupa* fraction [g]	Proportion of specimens found in the coarser fraction	Estimated number of specimens	Mean specimen width [mm]	Number of drilled specimens	Drilling Frequency	Mean maximum drillhole diameter [mm]	Mean minimum drillhole diameter [mm]
1	0	2.83	2.73	0.10	0.96	447	1.06	14	0.03	0.62	0.37
1	1	2.91	2.76	0.15	0.95	460	1.04	15	0.03	0.64	0.39
1	2	3.64	3.54	0.10	0.97	575	1.14	17	0.03	0.62	0.38
1	3	6.67	6.48	0.19	0.97	1054	1.14	15	0.01	0.61	0.38
2	0	9.92	9.54	0.38	0.96	1567	0.98	40	0.03	0.47	0.3
2	1	5.53	5.43	0.10	0.98	874	1.04	48	0.06	0.53	0.34
3	0	2.41	2.28	0.13	0.95	381	1.01	42	0.11	0.45	0.27
4	0	0.33	0.33	0.00	1.00	52	1.03	11	0.21	0.48	0.32
5	0	3.26	3.07	0.19	0.94	515	0.96	31	0.06	0.53	0.33
Total		37.50	36.16	1.34	0.95	5925	1.04	233	0.04	0.52	0.33

Specimen abundance estimated using the mean estimated specimen weight (0.00633 g). Mean per-sample frequency (i.e., grand mean of sample means) is 0.06, which is slightly higher than the value of 0.04 (0.039 if rounded to the third decimal place) reported below for total frequency.

### Qualitative and Quantitative Analyses of *Ditrupa arietina*

**Figure 4:**
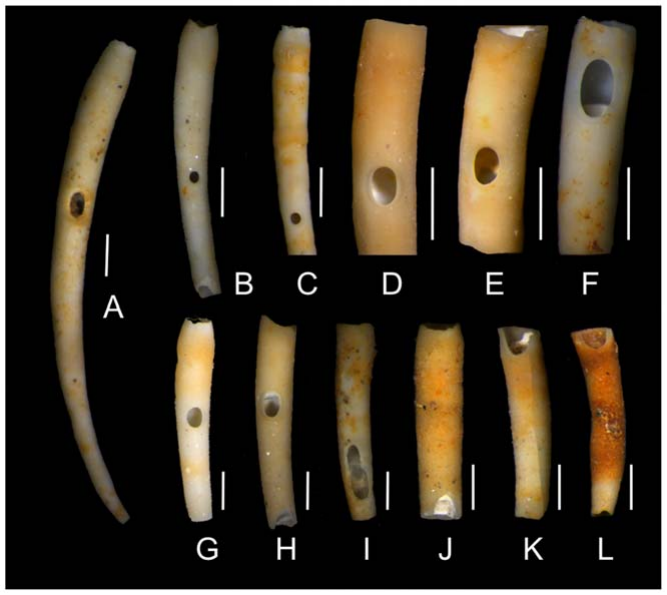
Drilled specimens of *D. arietina*. (a) A complete specimen with an oval drillhole located anteriorly; (b) A tube fragment with close-to-circular drillhole. Note a second drillhole partially preserved at the posterior end of the specimen (i.e., the tube broke across the drillhole); (c) A tube fragment with close-to-circular drillhole; (d–g) Tube fragments with singular complete oval drillholes; (h) A tube fragment with a complete oval drillhole (note the second, partially preserved drillhole located at the posterior end of the fragment); (i) An unusual tube fragment with two partly superimposed complete drillholes; (j–k) Tube fragments broken across drillholes. Scale bars are 2 mm for (a) and 1 mm for (b–k).

Specimens of *D. arietina* are dominated by fragments (Fig. 4b–l), but complete tubes that preserve both the anterior and posterior ends (Fig. 4a) are also present in the material. Some fragments preserve the anterior end (Fig. 4b–c,g–j), which makes it possible to estimate the maximum width of the tube for those fragments. Apart from fragmentation, tubes are well preserved: none of the hundreds of tubes and tube fragments analyzed under binocular showed any evidence of dissolution, abrasion, or bioerosion (other than drillholes). Encrustation is very rare and limited to encrusting foraminiferans, observed only in a few specimens. The tube surfaces appear unaltered and retain even minor morphological details such as concentric growth rings.

**Figure 5:**
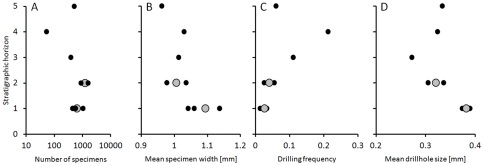
Stratigraphic changes in quantitative paleoecological patterns. (a) Abundance of specimens per standardize sample unit (207 g); (b) average specimen size (mean maximum specimen width); (c) drilling frequency (fragmentation-corrected proportion of specimens drilled); and (d) average drillhole size (mean minimum drillhole diameter). Solid small dots represent individual samples and larger gray dots are mean horizon values (arithmetic averages of sample values per horizon). Because horizons 3–5 are represented by one sample each, sample values also represent mean horizon values. See Table 2 for data summary.

Weight-standardized samples (207 g each) vary notably in estimated specimen abundance (Table 2), from 52 specimens (0.25 specimens per gram) in sample 4-0 to 1567 specimens in sample 2-0 (7.6 specimens per gram). In contrast, samples are highly consistent in terms of proportions of sieved fractions: all 9 samples are dominated by specimens recovered from >1 mm fraction (94 to 100% of total per-sample specimen mass; Table 2). Specimen abundance varies notably across horizons (reaching local minimum in Horizon 4), but is consistent across samples collected from within the same horizons (Fig. 5a). The short time-series (5 horizons) is insufficient to allow for a meaningful statistical evaluation of the observed temporal pattern (Fig. 5a).

Mean per-sample specimen body size, estimated by the maximum specimen width, varies in a narrow range (Fig. 5b), with mean width ranging from 0.96 to 1.14 mm. Variation in sample means across horizons is comparable to that observed within horizons.

### Morphometrics of Complete Specimens of *Ditrupa*

**Figure 6:**
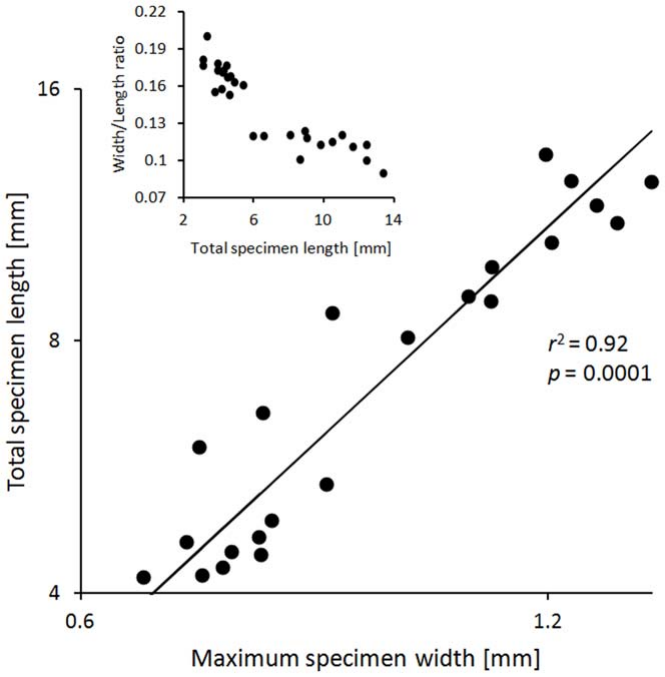
Bivariate scatter plot of total specimen length versus maximum specimen width based on 28 complete specimens. A solid line represents a reduced major axis regression model. Symbols: *r* ^2^ = coefficient of determination for the reduced major axis regression (associated *p*-value for the null hypothesis that the *r* ^2^ = 0). Inset: Bivariate scatter plot showing negative allometric relationship between specimen size (estimated here as total specimen length) and tube shape (expressed as width-to-length ratio). Small tubes tend to be twice as wide relative to their length when comparing with large tubes.

The complete specimens (Table S2) range from 3.1 to 13.4 mm in length (mean = 6.8 mm) and from 0.6 to 1.4 mm (mean = 0.9 mm) in width, respectively. A strong positive correlation between the length and width of the tubes is observed (Fig. 6). A reduced major axis regression suggests a strong allometric relation between the two tube dimensions given by the following allometric equation:
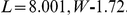
Where *L* is the total length of the complete specimen [mm] and *W* is the maximum width [mm]. The results indicate a strong allometry of specimen width relative to specimen length: the larger specimens tend to be relatively slimmer (width-to-length ratio of ∼0.1) than the smaller ones (width-to-length ratio ∼0.2) (Fig. 6, inset).

The strong length-width relation (*r* ^2^ = 0.92; Fig. 6) allows for deriving reliable estimates of specimen length for those tube fragments that can be measured reliably in terms of maximum width (i.e., specimens with anterior ends preserved), an advantage that is exploited below in evaluating spatial stereotypy in longitudinal distribution of the drillholes.

### Qualitative and Quantitative Analyses of Drillholes

In all 9 samples, specimens with drill holes were observed. The drill holes displayed an array of distinct features, including (1) regular, circular to elliptical outline; (2) larger outer diameter and smaller inner diameter; (3) penetration of only one tube wall; and (4) narrow size range of drill holes. Incomplete drillholes were not observed and none of the drillholes was repaired.

Multiple complete drillholes were observed in 9 out of 233 drilled specimens (3.9%): samples 2-0 (2 specimens), 2-1 (5 specimens), and 3-0 (2 specimens). One of the nine specimens included three complete drillholes, whereas the other eight had two drillholes each.

A total of 233 drilled specimens were identified (38 additional “drilled” specimens have been rejected after microscopic examination revealed that these holes were unlikely to represent drillholes, but physical damage to specimens). Using the mean estimated specimen weight, (*n*∼5925; see above and Table 2), an overall drilling frequency across pooled data is estimated at 3.9%. The maximum drilling frequency, when specimen number is estimated based on weight of large specimens (n∼3310; see above), is 7.0%. The minimum drilling frequency is 0.7%, when using weight of small specimens (n∼33098; see above). These end-point estimates suggest that the actual drilling frequency is unlikely to exceed 10% or be substantially less than 1%.

In addition, examination of end points of broken tubes under binocular microscope revealed that some of them broke across a drillhole (Fig. 4j–k). A quantitative assessment of one sample (Sample 3-0) revealed that 84 out of 221 tube fragments in that sample were broken across the drillhole. Moreover, 68 out of the 233 drilled specimens were broken across a second drillhole. Thus, drilling frequencies and frequency of multiple drill holes reported here represent a highly conservative estimate that must underestimate the actual drilling frequencies.

**Figure 7:**
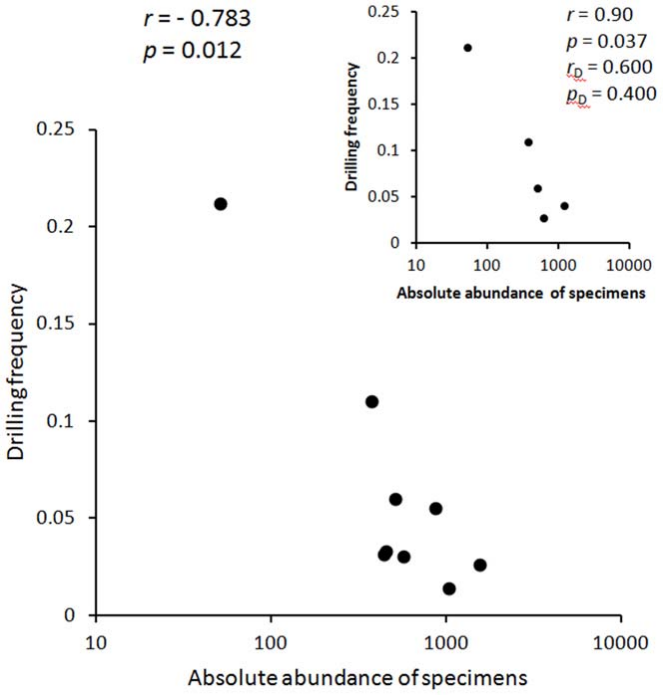
Bivariate scatter plot of absolute abundance of specimens plotted against drilling frequency. A significant negative correlation is observed, with drillholes being less frequent in samples that came from more fossiliferous units. Inset plot shows that the pattern holds when data are plotted by horizon (with values averaged across samples for Horizons 1 and 2), although first differences are not significantly correlated. Symbols, *r* = Spearman correlation coefficient; *r_D_* = Spearman correlation coefficient based on first differences; *p* = a two-tailed probability of *r* = 0; *p_D_* = a two-tailed probability of *r_D_* = 0.

Drilling frequencies vary notable across horizons (from 2.7% in Horizon 1 to 21% in Horizon 4), but is consistent across samples collected from the same horizons (Fig. 5c). The relative frequency averaged across samples (grand mean of sample means) is 6%. As in the case of specimen data, the short time-series (5 horizons) is insufficient to allow for a meaningful statistical evaluation of the observed temporal pattern (Fig. 5c). However, it is noteworthy that drilling frequency and specimen abundance are inversely related: the drilling frequency was lower in samples (Fig. 7) and horizons (Fig. 7, inset) that yielded more specimens per gram of sediment. This correlation is statistically significant for samples and for horizons (although, when horizon data are detrended, the trend ceases to be significant; Fig. 7, inset).

**Figure 8:**
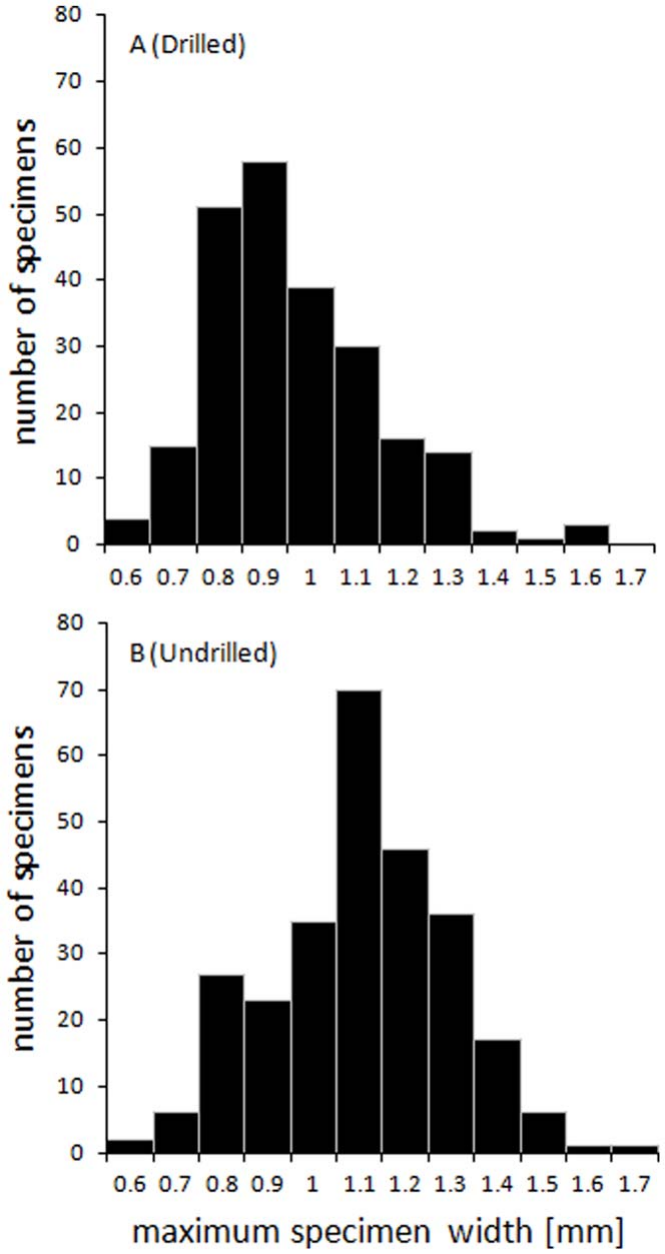
Size frequency distribution of *Ditrupa* specimens, with size estimated by the maximum specimen width. (a) Drilled specimens (*n* = 233); (b) Undrilled specimens (*n* = 270). See Table 3 (“pooled data” rows) for data summary and statistical tests.

**Figure 9:**
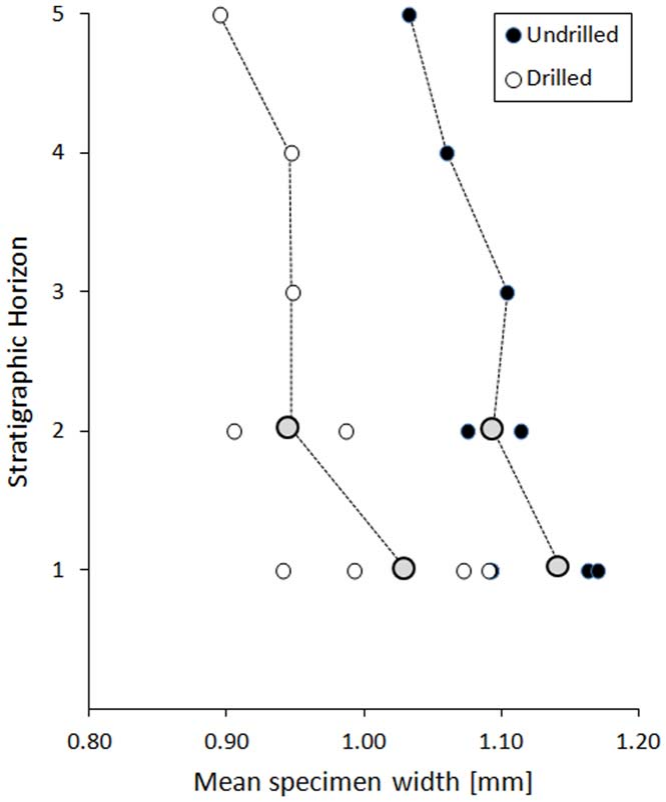
Comparison of mean specimen width for drilled and undrilled specimens grouped by stratigraphic horizon. For Horizons 1 and 2 multiple samples are available, whereas Horizons 3 through 5 are represented by one sample each. Small dots represent individual samples and larger gray dots are mean horizon values (arithmetic averages of sample values per horizon). See Table 3 for data summary and statistical tests.

**Figure 10:**
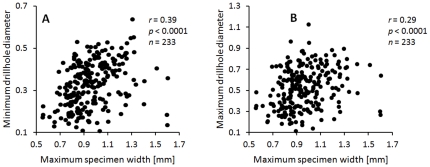
Correlation between specimen size (maximum width) and drillhole size. Drillhole size estimated as (a) minimum drillhole diameter, and (b) maximum drillhole diameter. Data pooled across all samples and horizons. Symbols, *r* = Spearman correlation coefficient; *p* = a two-tailed probability of *r* = 0; *n* = a number of specimens analyzed. See Table 4 for additional information.

Drilled specimens are significantly smaller than undrilled specimens. This pattern is observed for pooled data (Fig. 8; Table 3) and for each horizon analyzed separately (Fig. 9, Table 3). There is also a weak, but significant correlation between specimen size and drillhole diameter (Fig. 10, Table 4).

**Table 3:** Comparison of size frequency distributions for drilled and undrilled specimens.

Horizon	Sample	Specimen type	Number of specimens [n]	Mean width [mm]	Standard deviation of width [mm]	Maximum width [mm]	Median width [mm]	Minimum width [mm]	Wilcoxon 2-Sample Test*Z*-value[*p*-value]	Kolmogorov-Smirnov Test *D*-value[*p*-value]
1	0	undrilled	30	1.09	0.18	1.39	1.11	0.58	2.17^a^	0.51^a^
	0	drilled	14	0.99	0.14	1.25	0.94	0.84	[0.03]^a^	[0.01]^a^
	1	undrilled	30	1.09	0.15	1.38	1.11	0.77	2.64^a^	0.47^a^
	1	drilled	15	0.94	0.18	1.29	0.86	0.66	[0.008]^a^	[0.03]^a^
	2	undrilled	30	1.16	0.18	1.51	1.15	0.71	1.69	0.36
	2	drilled	17	1.09	0.19	1.61	1.04	0.83	[0.09]	[0.11]
	3	undrilled	30	1.17	0.17	1.59	1.19	0.79	2.28^a^	0.43
	3	drilled	15	1.07	0.14	1.32	1.03	0.91	[0.01]^a^	[0.05]
2	0	undrilled	30	1.07	0.14	1.31	1.07	0.83	4.19^b^	0.53^b^
	0	drilled	40	0.90	0.19	1.60	0.87	0.56	[<0.0001]^b^	[0.0001]^b^
	1	undrilled	30	1.11	0.21	1.47	1.09	0.63	2.80^a^	0.35^a^
	1	drilled	48	0.99	0.18	1.51	0.98	0.68	[0.005]^a^	[0.02]^a^
3	0	undrilled	30	1.10	0.23	1.69	1.11	0.74	2.86^b^	0.38^a^
	0	drilled	42	0.95	0.20	1.60	0.92	0.56	[0.002]^b^	[0.01]^a^
4	0	undrilled	30	1.06	0.20	1.40	1.07	0.77	1.63	0.40
	0	drilled	11	0.95	0.13	1.11	0.99	0.77	[0.10]	[0.15]
5	0	undrilled	30	1.03	0.22	1.51	1.06	0.67	2.50^a^	0.47^b^
	0	drilled	31	0.89	0.18	1.41	0.86	0.56	[0.006]^a^	[0.002]^b^
Pooled	--	undrilled	270	1.10	0.19	1.69	1.10	0.58	8.12^b^	0.39^b^
data	--	drilled	233	0.96	0.19	1.61	0.94	0.56	[<0.0001]^b^	[<0.0001]^b^

Specimen size estimated by maximum tube width [mm]. Basic descriptive statistics are reported here separately for drilled and undrilled specimens grouped by sample and for the pooled data (the last two rows). Non-parametric statistical tests for differences in central tendency (Wilcoxon 2-Sample Test) and shape of distributions (Kolmogorov-Smirnov Test) are also reported.

a Tests significant without Bonferroni correction at the assumed α = 0.05.

b Tests significant with Bonferroni corrections (α/number of tests; α = 0.005).

**Table 4:** Spearman correlation coefficients (upper right numbers) and corresponding *p*-values for the null hypothesis *r* = 0 (lower left numbers) for specimen and drillhole-derived variables.

Variable	Number of specimens	Drilling frequency	Maximum drillhole diameter[mm]	Minimum drillhole diameter[mm]	Maximum specimen width[mm]
Number of specimens	---	−0.783^a^	0.050	0.183	0.133
Drilling frequency	0.013^a^	---	−0.400	−0.467	−0.550
Maximum drillhole diameter	0.898	0.286	---	0.950^b^	0.717^a^
Minimum drillhole diameter	0.637	0.205	<0.0001^b^	---	0.767^a^
Maximum specimen width	0.732	0.125	0.030^a^	0.016^a^	---

Correlation based on mean per-sample values for the 9 samples for which quantitative data are available.

a Spearman coefficients significant at the assumed α = 0.05 significance value.

b Spearman coefficients significant at the Bonferroni-corrected α = 0.05/10 significance value.

**Figure 11:**
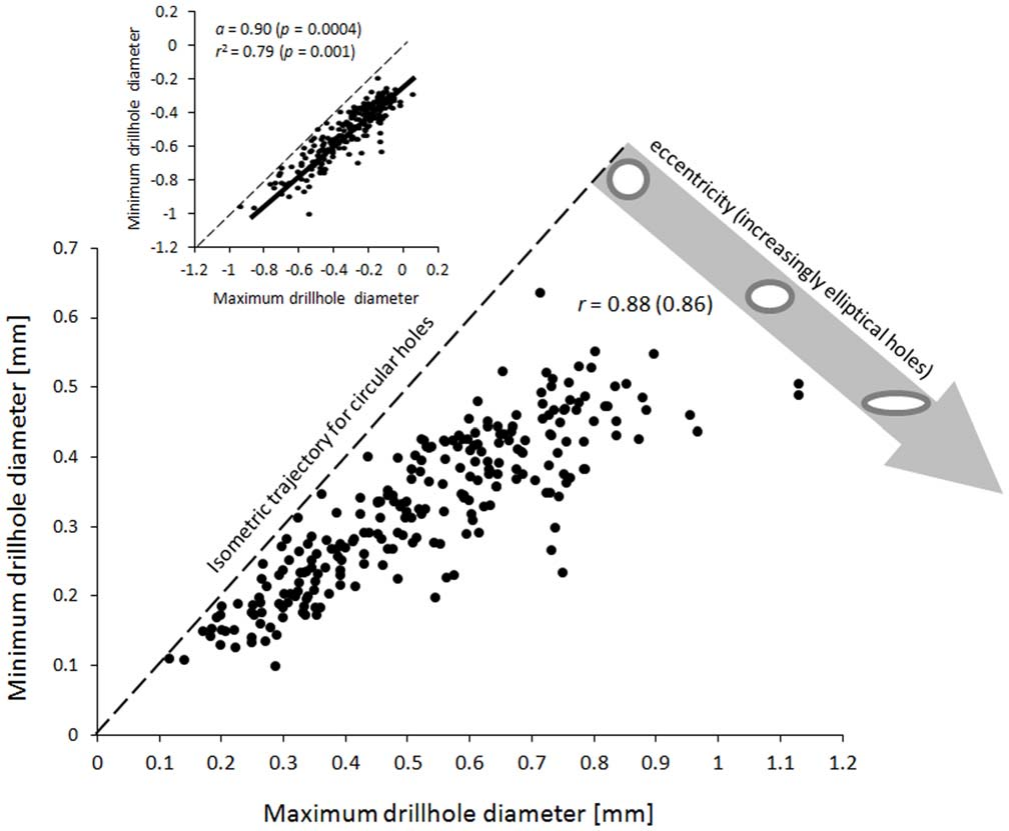
Bivariate scatter plot of maximum versus minimum drillhole diameter. Because minimum diameter cannot exceed maximum diameter, values above the diagonal line denoting perfectly circular holes of different sizes (isometric trajectory for circular holes) are not possible. Whereas a significant linear correlation exists between the two variables, the data are visually curvelinear, with larger drillholes displaying more notable departures from circularity (a wide grey arrow illustrates changes in shape of drillholes going away orthogonally from the isometric trajectory for circular holes). Symbols: r = Pearson's correlation coefficient (a value in parenthesis represents Spearman correlation coefficient). Inset: A bivariate plot of the same variable plotted in terms of log-transformed values. A solid line represents a reduced major axis regression model. Symbols: *a* = slope of the model (associated *p*-value for the null hypothesis that the slope value is *a* = 1), *r* ^2^ = coefficient of determination for the reduced major axis regression (associated *p*-value for the null hypothesis that the *r* ^2^ = 0).

**Figure 12:**
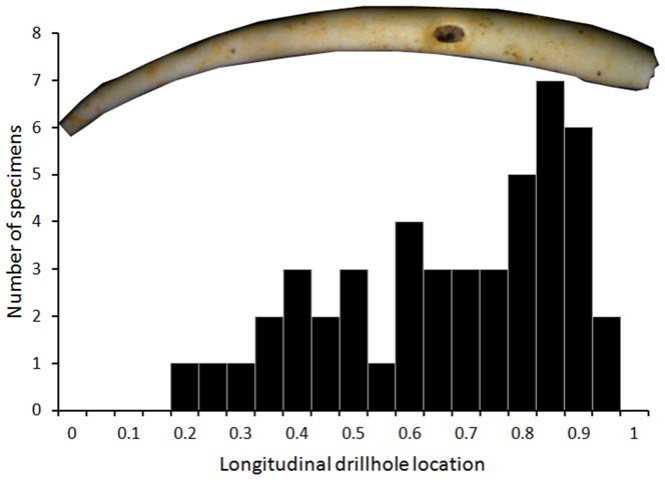
Longitudinal distribution of drillholes plotted for specimens with the anterior end preserved. Specimen length estimated using the allometric equation relating maximum width and length of complete specimens (see Fig. 6). Drillhole location is expressed proportionally as a distance of drillhole center from the anterior tube edge divided by the total estimated tube length. Thus, the value of 1 indicates a drillhole located at the anterior edge and value of 0.5 indicates a drillhole situated half way along the length of the tube.

Quantitative analysis of drillhole dimensions (Fig. 11, Table 4) indicate that drill holes vary from circular to strongly elliptical. The eccentricity of holes increases with drillhole size with large drillholes being almost invariably highly oval. This relation appears to follow an allometric trajectory as indicated by strong linearity of a log-log plot of the same data (Fig. 11, inset). The large, oval drillholes are invariably oriented with their longer axis parallel to the longer (posterior-anterior axis) of the tube (Fig. 4a, d–i). For specimens preserving their anterior end (those for which the tube width could be measured and the specimen length could be estimated), drillhole location can be estimated quantitatively. The results suggest that drillholes concentrate anteriorly (Fig. 12).

## Discussion

### Paleoecology and Taphonomy of *Ditrupa arietina*

The systematic sampling summarized above indicates that *Ditrupa arietina* is a dominant, occasionally exceedingly abundant, bioskeletal component of the studied Pliocene succession. This is consistent with ecological observations on recent *D. arietina* which occurs in heavily populated patches, with densities reaching hundreds or even thousands of individuals per square meter of seafloor [55], [61]. Thus, the abundant presence of *D. arietina* tubes in the Cabo Cape succession may not only reflect taphonomic conditions favorable for preservation of bioskeletal materials, but also opportunistic ecology that typifies free-living, tube-dwelling polychaetes today. It is noteworthy that previous paleontological reports on fossil occurrences of free-living serpulids also highlight their exceptionally high abundance [41].

The Cabo Cape lithology, sedimentary structures, and associated macrobenthic fauna consistently suggest that the studied units record a succession of open marine, soft-bottom, depositional settings, with variable sedimentation rates. This depositional setting resembles closely present-day habitats in which *Ditrupa* thrives. Unlike other serpulids, the recent *Ditrupa* is a free-living epifaunal to semi-infaunal polychaete found in muddy to fine sandy sediments of continental shelves in areas characterized by high sedimentation rates and turbulent waters. In such settings *Ditrupa* can form exceedingly dense populations [37], [45], and its tubes often constitute the main component of the biogenic carbonate sediments [62]. Although the exact bathymetric history of Cabo Cope succession cannot be readily reconstructed, it should be noted that habitats dominated by *Ditrupa* populations can be found today over a wide range of depths, from 20–30 m along the western Mediterranean coast [36], [63] to 100–250 m in the Açores [49], and 300 m around Iceland [45]. In sum, the abundant occurrence of *D. arietina* reported here is consistent with neontological observations.

The near complete absence of encrustration reported above is notable given that diverse epibionts have been documented as colonizers of recent *Ditrupa*. For example, Sanfilippo [45] noted that tubes of *D. arietina* from Iceland were often densely colonized by brachiopods, solitary scleractinians, barnacles, serpulids, bryozoans, sponges, and foraminiferans. Moreover, most tubes of dead specimens were secondarily inhabited by sipunculids. The colonization by epibionts and the occupation of empty tubes by sipunculids were also reported for Azorean populations of *D. arietina* [49]. In contrast, the near complete absence of encrusters in Cabo Cope samples (except for a few specimens encrusted by foraminiferans) may either reflect a partly infaunal mode of life of *D. arietina*, or quick burial after death, or combination of those factors. Whether any of the studied specimens was affected by secondarily sipunculid colonization [49] is difficult to evaluate because no diagnostic trace fossils induced by sipunculid colonization have been documented so far to our knowledge.

The tight correlation between tube length and tube width, with all specimens following a single allometric trajectory suggest that all specimens analyzed here represent a single species. This reaffirms our qualitative assignment of all specimens into a single species (see section “Taxonomic Identity and Morphology of Sampled Serpulid Polychaetes” above).

### Ichnotaxonomy, Ethology, and Taphonomy of Drillholes

Based on circular outline and parabolic vertical profile, the drillholes documented here are classified to the ichnospecies *Oichnus paraboloides* Bromley, 1981 [64], [65]. This is despite the fact that many of the holes are elliptical rather than circular in outline, as is the case for *Oichnus ovalis* Bromley, 1993 [66]. However, note that the longer axis of an oval hole always parallels the longitudinal axis of a tube (see also fig. 1 in [43] and fig. 3 in [46]). Thus, the pronounced eccentricity of some of the holes is likely a mere geometric artefact that is expected for circular borings drilled by the boring organ which is constrained by tube curvature [43], [46]. This artefact is increasingly pronounced for larger holes (Fig. 11), where the effect of tube curvature is more pronounced.

When found in bioskeletal remains, *Oichnus* ichnospecies tend to be attributed to predatory activity, or *praedichnia* [67]. This interpretation can be assessed using multiple lines of evidence. In our case, biological origin is suggested by drillhole morphology: holes display regular outline, are perpendicular to the tube surface, and their outer diameter tends to exceed their inner diameter (suggestive of penetration from outside). Moreover, drillholes invariably penetrate only one side of the tube (note: substrate borings often penetrate throughout multiple walls of a shelly organism; [68], [69]) and are either singular or limited to, at most, a few borings. Non-random (anterior) distribution of drillholes, a relatively narrow range of drillhole sizes, and a weak (but statistically significant) correlation between drillhole diameter and tube size further support the interpretation that drillholes record live-live interactions between *D. arietina* and a drilling organism.

It is difficult to establish the identity of drilling predators (or parasites) responsible for drillholes. *O. paraboloides* has been frequently attributed to carnivorous naticid gastropods, which prey on a variety of benthic organisms (mostly mollusks) in soft-substrate habitats. However, many organisms produce drillholes, including octopods, nematods, and multiple clades of gastropods [2]. None of the taxa reported from Cabo Cope (Table 1) belongs to a group of known drilling predators (non-drilling predators are present in Cabo Cope, including five species of scalariid gastropods, which today prey primarily on coelenterates). However, the common drilling organisms known today either lack biomineralized skeletons or secrete aragonitic shells, which are not preserved in the Cabo Cope fossil assemblages.

In previous studies dealing with drilling predation on *Ditrupa*, naticid and muricid gastropods have been postulated as the most likely culprits. Sanfilippo [45] attributed predation on Pliocene *Ditrupa* tubes from Italy to the activity of two groups of carnivorous gastropods: naticids for contersunk holes (morphologically analogous to the borings reported here) and muricids for smaller, cylindrical ones. Morton and Harper [43] tentatively attributed drillholes in Recent *D. arietina* from the Azores to the predatory activity of *Natica prietoi*, a small naticid species co-occurring with *D. arietina*. Similarly, Klompmaker [46] attributed drillholes found in *D.* cf. *arietina* to naticid predation. Drillholes found in fossil scaphopod shells, which are similar to *Ditrupa* in terms of morphology, body size, and mode of life, have been also attributed to the activity of naticid gastropods [70], [71]. It is possible that drillholes reported here were also produced by naticid gastropods, but existing data do not allow for a reliable assessment of drilling organisms that produced those trace fossils.

Multiple drillholes are infrequent (3.9%; although this value is likely underestimated; see below). Nevertheless, the presence of multiple complete drillholes (2 or 3) suggests that predators failed on occasions; successful predatory attacks typically involve one drillhole only [72]. However, in this case, predator's failure is unlikely to have been caused by active escape response from this immobile prey, but rather by other physical or biotic disturbances. A nearly identical frequency of multiple drill holes (3.4%) has been reported recently for Pliocene *Ditrupa* from the Netherlands [46].

Whereas multiple line of evidence suggest that the drillholes record live-live interactions, it is theoretically feasible that drillholes were made by predators preying on sipunculids that are known today to be a secondary colonizer of empty *Ditrupa* tubes. However, in recent tubes from Azores some of the drilled tubes were inhabited by sipunculids [49], suggesting that, at least in some cases, drillholes have been made prior to colonization events.

Finally, the fact that tubes are often broken off across a drillhole has two interesting taphonomic corollaries. First, such breakage patterns suggest that estimates of drilling frequencies are inherently biased [73] because drilled specimens are less likely to be preserved with a drillhole intact. Second, drilling induces fragmentation of tubes lowering the quality of the fossil record for tube dwelling serpulids. Biologically-facilitated fragmentation of this type has been reported previously in other settings (e.g., worm borings in brachiopod shells [74]). Frequent breakage across drillholes has been reported recently for Pliocene *D.* cf. *arietina* from the Netherlands, including discussion of potential biases [46].

### Drilling Patterns

Drillholes have been reported by several authors for both recent [43], [49] and fossil [44], [45], [46] *Ditrupa*. However, quantitative data are limited, with notable exceptions of detailed documentations by Morton and Harper [43] and Klompmaker [46].

Drilling frequencies reported here – varying across samples from 2.7% to 21% (and, as discussed above, these estimates likely underestimate the actual drilling frequencies) – are well within the range of drilling frequencies observed in the Cenozoic fossil record of mollusks, echinoids, or brachiopods (see compilations in [3], [18], [19], [34]). Comparing with previous studies on *Ditrupa*, our estimates are notably higher than an estimate of 1.9% reported for recent Azorean *D. arietina* [43], lower than fragmentation-corrected estimates for *D.* cf. *arietina* (18.6–62.1%) from the Pliocene of the Netherlands [46], and notably lower than the very high frequency of 65% reported for *Ditrupa* sp. from the Eocene of the Paris Basin [44].

Despite notable differences in drilling frequencies, the results reported here share multiple similarities with previous studies. First, the absence of repaired or incomplete holes and the presence of specimens with two or three borings have been also reported by Morton and Harper [43] (although sporadic incomplete drillholes have been reported from the Pliocene of the Netherlands [46]). Thus, drillers appear to display high prey effectiveness in terms of drillhole completeness, but failed attempts are suggested by multiple drillings in single prey specimens. Second, as in the case of recent Azorean *D. arietina* [43], the drilled specimens from Cabo Cope are significantly smaller than undrilled specimens, both for pooled data and for each horizon separately (Figs. 8–9). This pattern may either reflect size-selective predation or different post-mortem history of drilled and undrilled tubes. Third, drillhole diameters are remarkably similar for the Cabo Cope specimens (range: 0.58–1.69 mm), Pliocene *Ditrupa* cf. *arietina* form the Netherlands (range: 0.6–2.0 mm; [46]) and the recent Azorean samples (outer diameter up to 0.7 mm; [43]). Fourth, drillholes in *Ditrupa* tend to be distributed non-randomly in terms of their location on the tube [43], [45], although the previous studies all documented preferential occurrences of drillholes in the middle part of the tube, regardless of the tube length, whereas our data (Fig. 12) suggest the anterior site-stereotypy.

In addition to the above quantitative patterns, we also found a positive significant correlation between drillhole diameter (a putative proxy for predator's size) and tube length. This weak correlation may either reflect a behavioral relationship (bigger predators attack bigger prey) or a taphonomic bias (big holes drilled in small tubes facilitate fragmentation).

Given morphological and ecological similarities between *Ditrupa* and scaphopod mollusks, it is noteworthy that scaphopods have been also known as prey of drilling organisms [70], [71], [75], and some remarkable similarities may exist between drilling patterns in the two prey groups [46]. As in the case of *Ditrupa*, drilling frequencies vary notably across case studies. For example, Li et al. [70] reported a drilling frequency of 35% for *Dentalium gracile* from the Upper Cretaceous of Manitoba (Canada), whereas only 1% of complete drillholes was reported for *Fissidentalium* sp. from the Miocene of the Netherlands [71]. In contrast to *Ditrupa*, incomplete borings are observed in scaphopods, with as much as 50% of drillholes representing failed attempts [71].

The limited number of detailed case studies on drilling predation on free-living tube dwelling serpulids, and comparably scarce treatment of ecologically and morphologically similar scaphopod mollusks, make it difficult to offer any far reaching generalizations about the importance and nature of predator-prey interactions for those prey groups.

### Final Remarks

The quantitative survey of the Cao Cope marine succession (Pliocene, Spain) revealed abundant accumulations of calcitic tubes of the free-living serpulid polychaete *Ditrupa arietina*. In all studied horizons, tubes drilled by an unknown drilling organism were present, with drilling frequencies ranging from 2.7 to 21%. Drillholes (*Oichnus paraboloides*) were always complete and never repaired, but some specimens contained two or more drillholes. The drillhole size, drillhole morphology, and non-random distribution of drillings are consistent with previous reports on *Ditrupa* and reveal multiple parallels with drilling patterns documented for ecologically and morphologically similar scaphopod mollusks. This and other recent studies suggest consistently that the present-day populations and fossil assemblages of free-dwelling serpulid polychaetes represent a viable source of data on drilling predation and related ecological and paleoecological data on soft-bottom, marine communities.

## Supporting Information

Table S1 A summary of specimen-level numerical measurements for all drilled specimens and a random sample of non-drilled specimens of *Ditrupa arietina* from the Pliocene of Spain. A total of 503 specimens are included in the dataset. However, because 10 additional drillholes are recorded for specimens with multiple drilling, the table includes 513 rows of data. The extra rows are identified by value = “0” for the column “Count”. The column labels (first row, from left to right) are as follows: **W_1** – weight of 1 mm fraction of the sample [grams]; **W_0.5** – weight of 0.5 mm fraction of the sample [grams]; **N_min** –minimum sample size, assuming average specimen weight of 0.01133 g [count]; **N_avg** – average sample size, assuming specimen weight of 0.00633 g [count]; **N_max** – maximum sample size, assuming specimen weight of 0.00133 g [count]; **D** – Number of drilled specimens [count]; **D_min**, **D_avg**, **D_max** – minimum, average, and maximum drilling frequencies based on N_min, N_avg, and N_max, respectively [proportion]; **Spec** – specimen ID number; **MaxD** – maximum drillhole diameter [mm]; **MinD** – minimum drillhole diameter [mm]; **Diam** – specimen diameter (maximum diameter of Ditrupa tube) [mm]; **Drilled** – specimen drilled = 1, specimen undrilled = 0; **Count** – specimen = 1, additional entry for specimens with multiple drillholes = 0; and **Ndrill** – Number of drillholes per specimen [count]. The file was last updated on March 2, 2012.
(XLS)

Table S2 A summary of bivariate morphometric measurements for 28 complete specimens of *Ditrupa arietina* from the Pliocene of Spain. The column labels (first row, from left to right) are as follows: **Specimen** – specimen number; **Length** – specimen length [mm]; and **Width** – specimen maximum width [mm]. The file was last updated on March 8, 2012.
(XLSX)
